# Reflection on a professional advisory group to inform the use of patient empowerment tools within an implementation science research project

**DOI:** 10.1186/s41687-024-00811-y

**Published:** 2024-12-02

**Authors:** Katherine E. Woolley, Nia J. Jones, Robert Letchford, Kathleen L. Withers

**Affiliations:** 1 CEDAR, Cardiff and Vale University Health Board, Cardiff, UK; 2 Physiotherapy Department, Cardiff and Vale University Health Board, Cardiff, UK; 3 Welsh Value in Health Centre, The Hub, Unit 2, Gwaun Elai Medi Campus, Llantrisant, CF72 8XL UK

**Keywords:** Patient participation, Patient activation, Patient and public involvement, Professional advisory group, Stakeholders, Proposal development

## Abstract

Professional advisory groups, with patient and public involvement (PPI) representatives, can be used for co-production within research projects. This paper aims to document the benefits and challenges of undertaking stakeholder and participant engagement for an implementation research project within NHS (National Health Service) Wales. A patient focused research project, initiated by clinicians, on the use of patient empowerment tools within standard patient care, used a professional advisory group to identify appropriate tools to use within the research proposal. The professional advisory group was made up of therapists, NHS stakeholders, academics and PPI representatives. A hybrid-meeting style was employed to optimise participation for all members of the group. Benefits of the professional advisory group included increased engagement and ownership of the study due to co-creation, and obtaining important contextual information and lived experience. However, challenges included keeping the discussion on topic due to pre-conceived agendas, pleasing everyone in the room due to varied backgrounds, and technological issues. Future professional advisory groups should consider how to facilitate the full involvement of PPI representatives within the discussion and having a variety of resources to present the topic of discussion. Furthermore, clearly communicating what the purpose and direction of the research project is and how it fits into the wider system, should be carefully considered. Overall, it was recognised that the professional advisory group was of significant value to shape the research proposal. Due to the situational challenges faced by healthcare professional within the NHS and preconceived ideas for solutions, it is hoped that by involving stakeholders early in the process there will be greater acceptance and usability of the research findings.

## Background

Research can become very rooted in process, such as obtaining ethical approval, identification of clinical sites to target, data management and storage procedures and dissemination. There is an increasing focus for health service improvements and research to include co-production and involve stakeholders and participants from project conception to completion.

A range of co-production and involvement methodologies are available to researchers, which can include the public as instigators, co-designers and co-implementors, all of which have benefits and challenges [1]. Although public involvement and co-production is not a new process, with the first patient groups being formed in the 1950s [2], there is a paucity of evidence on the use of professional advisory groups within an implementation science project for the National Health Service (NHS).

The purpose of this paper is to discuss the benefits and challenges of undertaking stakeholder and participant engagement for an implementation research project within the NHS.

## Setting

An implementation science research project is being undertaken within Cardiff and Vale University Health Board, to inform the application of patient empowerment tools into practice within two clinical areas. The results of this project hope to inform future research to be scaled and spread nationally across Wales, to create a standardised approach.

This project was initiated by clinicians from physiotherapy in a patient focused approach, after identifying a current variety of uses of patient empowerment tools and challenges in their implementation in clinical practice. Patient empowerment tools are short questionnaires that are designed to measure a patient’s level of motivation, engagement and capacity to understand their care [3]. Clinicians need to understand the level of engagement their patients have with their care, in order to tailor the care they provide appropriately. With the ultimate aim of patient empowerment is to shift towards promoting self-management behaviours, within a values-based approach to healthcare delivery.

As part of the project development, patients and end-users (healthcare staff) were involved as co-designers by attending a professional advisory group. The aim of the professional advisory group was to identify which tools to explore for their use within the two clinical areas. The group comprised of therapists, NHS stakeholders, academics and patient and public involvement (PPI) representatives. It should be noted that generalisability of the group’s composition was limited to those therapies departments within a multicultural urban setting within NHS Wales (podiatry and physiotherapy) that were keen to deploy patient empowerment tools, but did not extend to other staff groups (e.g., occupational therapy, speech and language, dietician) or multiple health board across Wales.

The purpose of the group was to discuss a range of tools (six patient empowerment tools and two health literacy tools) identified from a rapid literature review [4] to select the most generalisable and acceptable tools to explore within the clinical setting. The tools were discussed two at a time, to identify positive and negative features (e.g., length, appropriateness of questions, usability in setting), in the following groups: Health Literacy, Patient Activation, Long-term Conditions, and Self-management Needs and Behaviours. Post-it notes and hard copies of the tools were available for participants to write notes and thoughts onto. At the end of the session, all the patient empowerment tools were ranked individually by each participant, followed by a group ranking. These rankings were then used by the researchers to determine the three patient empowerment tools to use as part of the research project. The meeting was hybrid, with five members attending in person and three members via Microsoft Teams.

## Challenges of undertaking professional advisory groups

The composition of a professional advisory group is key to obtain the lived experience [5]. As this project came about due to real-world constraints and differing views on solutions to overcome the barriers, there were contested problem definitions, which created a complex problem [6]. By nature of the topic being a complex problem there was a challenge to keep on topic to achieve the objectives of the meeting due to pre-conceived ideas. These preconceived ideas included: preferences on which tool to implement, how the tools should be implemented, how and when the tools should be used. Each individual professional had their own motivating factors for attending the group and made an important contribution in deciphering practical solutions to a complex problem. However, it was essential to manage these within the aims of the project and manage attendee’s expectations (e.g., coming to a mutual agreement, which may not be their original preconceived idea).

On the flip side, clinical experience and perception had to be balanced against the methodological rigour of the research proposal, which was achieved by having academic representation. However, this led to methodological scrutiny of the pragmatic approaches taken during the rapid literature review, where tools were ranked against the Psychometric and Pragmatic Evidence Rating Scale (PAPERS) grading criteria rather than COnsensus-based Standards for the selection of health status Measurement Instruments (COMSIN) due to the greater consideration of pragmatic qualities rather than just the traditional evidence-based assessment and methodological quality of studies [7]. Therefore, the rapid literature review methodology had to be defended, in the most practical way possible, to assure clinical and lay members in the group, especially those with preconceived ideas, that the evidence provided was reliable.

Although, it is very important for processes and methods to be scrutinised, it can be a challenge for patient representatives to participate in the conversation, especially when the lines of discussions use of highly complex medical and scientific terminology. However, a learning point would be to have a member of the research team assisting each lay participant to help them follow the discussion and direct them to the correct tools at the appropriate time for example. Consideration should be given to PPI members attending online, by using for example, the chat or screen sharing function.

Interestingly, Bell et al. [8] discussed the role of group dynamics and how it impacts the discussion and outcomes of a meeting. This should be taken into consideration when reporting outputs, especially PPI participants that are actively engaged their own health [5]. It is important to note that the dynamic of this group was altered by technological issues, meaning that online attendees struggled to hear all of the conversation in room. Although an assessment of the true impact of the technological issues on a hybrid meeting could not be undertaken, it is highly likely that some of the difficulties experienced by participants accessing the meeting online could have been resolved more easily without this interference. Further research would be required to assess potentially how group dynamics change when faced remote and hybrid meetings as well as the impact of technological issues.

## Impact of professional advisory groups

The whole purpose of involving stakeholders early on in the research process is to understand stakeholder needs and priories, current facilitators and barriers and balance what the evidence base says against practicality and feasibility. In this case some measures were shown to be valid within the literature but were not necessarily relevant to the setting of interest. Although Gray-Burrows et al. [9] found contested evidence on the use of PPI within informing the intervention context, patient empowerment tools are not dissimilar to surveys, for which there is consensus of the role of PPI. Within this professional advisory group the patient representatives were particularly valuable in providing insights into what their thoughts may be if presented with the tools in a clinical setting and how acceptable the tools were to their care. However, the real value of the professional advisory group was obtaining the lived experience and increasing engagement from stakeholders including patient representatives. Discussion that revolved around lived experience brought to attention the rationale for the research project and provided crucial contextual information to the researchers. Furthermore, by the end of the session it felt that the stakeholders had a greater understanding of the purpose and direction of the research proposal, and it is hope that by being involved within the proposal development there would be greater engagement and support with data collection and acceptance of the study findings as well as ensuring that the project aligns with local and national priorities.

## Lesson learned and concluding remarks

This reflection has added to the existing evidence base for the value of patient representation and stakeholder engagement within implementation science. However, it has highlighted the requirement for effective communication within both the project and how the evidence was generated across multiple knowledge and engagement levels, to reduce the impact of preconceived ideas. Consideration may need to given to the provision of different ways of presenting the same information such as a detailed academic piece, a lay summary or even a short lay video for patient representatives. Furthermore, using an implementation framework, such as, The Behaviour Change Wheel [10] or Exploration, Preparation, Implementation, Sustainment (EPIS) framework could help explain how this piece of planned research fits into the wider context, and what it hopes to achieve, but also what it may not answer. Through improvement and creating innovative ways of communicating the research project with both patient representatives and wider stakeholders, it is hoped that future meetings will keep close to the topic at hand and increase both the confidence in the researchers and the conclusion drawn from the activity.

## Data Availability

Not applicable.

## Abbreviations

NHS: National Health Service
PPI: Patient and Participant Involvement
